# Pollen Allergens for Molecular Diagnosis

**DOI:** 10.1007/s11882-016-0603-z

**Published:** 2016-03-22

**Authors:** Isabel Pablos, Sabrina Wildner, Claudia Asam, Michael Wallner, Gabriele Gadermaier

**Affiliations:** Department of Molecular Biology, University of Salzburg, Hellbrunner Straße 34, 5020 Salzburg, Austria; Christian Doppler Laboratory for Biosimilar Characterization, University of Salzburg, Hellbrunner Straße 34, 5020 Salzburg, Austria

**Keywords:** Pollen allergens, Molecule-based diagnosis, Grass pollen allergens, Tree pollen allergens, Weed pollen allergens

## Abstract

Pollen allergens are one of the main causes of type I allergies affecting up to 30 % of the population in industrialized countries. Climatic changes affect the duration and intensity of pollen seasons and may together with pollution contribute to increased incidences of respiratory allergy and asthma. Allergenic grasses, trees, and weeds often present similar habitats and flowering periods compromising clinical anamnesis. Molecule-based approaches enable distinction between genuine sensitization and clinically mostly irrelevant IgE cross-reactivity due to, e. g., panallergens or carbohydrate determinants. In addition, sensitivity as well as specificity can be improved and lead to identification of the primary sensitizing source which is particularly beneficial regarding polysensitized patients. This review gives an overview on relevant pollen allergens and their usefulness in daily practice. Appropriate allergy diagnosis is directly influencing decisions for therapeutic interventions, and thus, reliable biomarkers are pivotal when considering allergen immunotherapy in the context of precision medicine.

## Introduction

Allergic reactions to pollen represent the most frequent type I allergies affecting up to 30 % of the industrialized population (www.eaaci.org). Climatic changes are expected to influence the duration as well as the intensity of pollen seasons which might in hand with air pollution contribute to increased numbers of respiratory allergy and asthma [1, 2]. In addition, pollen-derived nanovesicles and other small components (e.g., adenosine) may play a specific role in the course of allergic diseases [3, 4]. Clinical anamnesis of pollen allergies to identify the disease-eliciting source can be hampered by similar habitat and flowering periods of certain plants and the fact that symptoms may be elicited by pollen transported far by the wind [5]. In addition, patients are often multi-sensitized to diverse allergen sources due to IgE cross-reactivity, co-sensitization, or both [6–10]. Skin prick tests and specific IgE detection using crude pollen extracts are currently performed in routine allergy diagnosis. Allergen extracts however contain a variety of allergenic and non-allergenic components, and standardization of pollen extracts is difficult due to varying source material and product preparations [11]. Besides source specific and thus genuine marker allergens, also minor allergens including panallergens as wells as allergens with cross-reactive carbohydrates (CCDs) are present in diagnostic extracts which might impede exact diagnosis. Molecular diagnosis using well-characterized purified allergen components from natural source or produced as recombinant molecule allows clinicians to obtain detailed information on sensitization profiles and thus supports improved patients’ management [11, 12•].

**Fig. 1:**
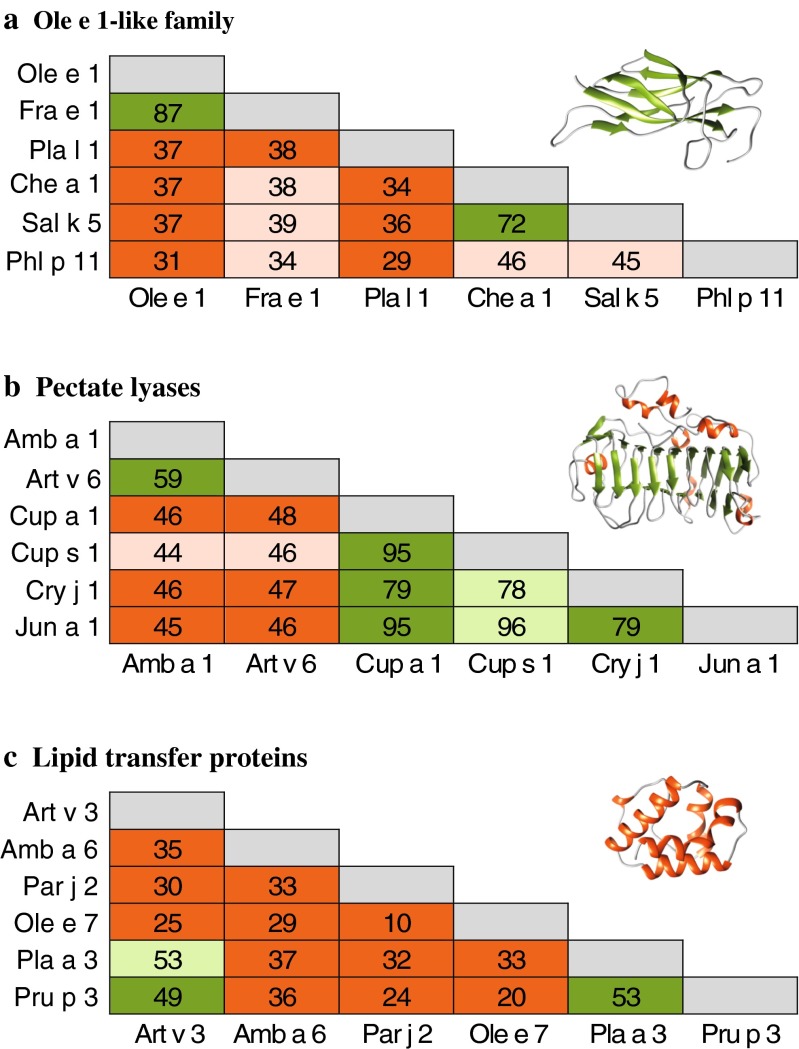
Sequence identity matrix and 3D-models of allergenic protein families. **a** Ole e 1-like proteins and structure of Pla l 1.0101 (4Z8W), **b** pectate lyases and model of Amb a 1.0101 (template 1PXZ), and **c** lipid transfer proteins and model of Art v 3.0201 (template 2B5S). Multiple sequence alignments performed in Clustal Omega. Models were generated using Swiss-Model (www.swissmodel.expasy.org), and ribbon cartons are shown using UCSF Chimera (www.cgl.ucsf.edu/chimera). *Green boxes* represent demonstrated IgE cross-reactivity, *light green boxes* represent potential IgE cross-reactivity based on high sequence identity, *red boxes* represent no/limited demonstrated IgE cross-reactivity and *light red boxes* represent no/limited IgE cross-reactivity based on low sequence identity

More than 150 pollen allergens are officially acknowledged by the IUIS allergen nomenclature sub-committee originating from grasses, trees, and weeds (www.allergen.org). This review focuses on the most relevant and commercially available components which are discussed in more detail (Tables 1 and 2). We also provide an overview on diverse IgE cross-reactivity profiles within the Ole e 1-like, pectate lyase and non-specific lipid transfer protein (nsLTP) families (Fig. 1).

**Table 1:** Grass pollen allergens

	Flowering period	Grass group 1 (Beta-expansin)	Grass group 2 (expansin-like protein)	Grass group 3 (expansin-like protein)	Grass group 4 (Berberine-bridge enzyme)	Grass group 5 (Ribonuclease)	Grass group 6	Polcalcin	Ole e 1-like protein	Profilin	Grass group 13 (Polygalacturonase)
Timothy grass	5–8	*Phl p 1* ^a^	*Phl p 2* ^a^	*Phl p 3* ^b^	*Phl p 4* ^a^	*Phl p 5* ^a^	*Phl p 6* ^a^	Phl p 7^a^	Phl p 11^a^	Phl p 12^a^	*Phl p 13*
*Phleum pratense*											
Perennial ryegrass	5–8	*Lol p 1*	Lol p 2	*Lol p 3*	Lol p 4	*Lol p 5*			*Lol p 11*		
*Lolium perenne*											
Orchard grass	5–6	*Dac g 1*	Dac g 2	*Dac g 3*	*Dac g 4*	*Dac g 5*					
*Dactylis glomerata*											
Kentucky blue grass	5–8	*Poa p 1*				*Poa p 5*					
*Poa pratensis*											
Bermuda grass	all season	*Cyn d 1* ^a^						Cyn d 7		Cyn d 12	
*Cynodon dactylon*											
Bahia grass	all season	*Pas n 1*									
*Paspalum notatum*											
Johnson grass	all season	*Sor h 1*	Sor h 2								Sor h 13
*Sorghum halepense*											

Major allergens highlighted in italics

^a^Available for single- and multiplex analysis

^b^Allergen not officially acknowledged by the WHO/IUIS allergen nomenclature sub-committee

**Table 2:** Relevant tree and weed pollen allergens

	Flowering period	Bet v 1-like protein (PR-10)	Profilin	Polcalcin	Phenylcoumaran benzylic ether reductase-like	Polygalacturonase	Plant invertase/pectin methylesterase inhibitor	1,3 beta-glucanase (PR-2)	Pectate lyase	Defensin-prolin fusion (PR-12)	nsLTP (PR-14)	Ole e 1-like protein	Pectin methylesterase	Cysteine protease
TREE POLLEN														
Birch	3–4	*Bet v 1* ^a^	Bet v 2^a^	Bet v 3	Bet v 6^c^									
*Betula verrucosa*				Bet v 4^a^										
Alder	2–3	*Aln g 1* ^b^		Aln g 4										
*Alnus glutinosa*														
Hornbeam	4–5	*Car b 1*												
*Carpinus betulus*														
Hop-hornbeam	4–5	*Ost c 1*												
*Ostrya carpinifolia*														
Hazelnut	2–3	*Cor a 1* ^b^	Cor a 2		Cor a 6									
*Corylus avellana*														
Beech	4–5	*Fag s 1*												
*Fagus sylvatica*														
Chestnut	5–6	*Cas s 1*												
*Castanea sativa*														
Oak	4–5	*Que a 1*												
*Quercus alba*														
London plane tree	4–5					*Pla a 2* ^b^	*Pla a 1* ^a^				Pla a 3^b^			
*Platanus acerifolia*														
Olive	4–6		Ole e 2	Ole e 3	Ole e 12^d^			*Ole e 9*			*Ole e 7* ^a^	*Ole e 1* ^a^	*Ole e 11*	
*Olea europea*				Ole e 8				*Ole e 10* ^e^						
European ash	3–5											*Fra e 1*		
*Fraxinus excelsior*														
Common privet	6–7											*Lig v 1*		
*Ligustrum vulgare*														
Lilac	4–5			Syr v 3								*Syr v 1*		
*Syringa vulgare*														
Mediterranean cypress	1–2								*Cup s 1*					
*Cupressus sempervirens*														
Arizona cypress	8–9								*Cup a 1* ^b^					
*Cupressus arizonica*														
Japanese cypress	1–2					*Cha o 3*			*Cha o 1*					
*Chameocyparis obtuse*														
Japanese cedar	2–3					*Cry j 2*			*Cry j 1* ^b^					
*Cryptomeria japonica*														
Mountain cedar	12–1					*Jun a 2*			*Jun a 1*					
*Juniperus ashei*														
WEED POLLEN														
Ragweed	7–9		Amb a 8	Amb a 9					*Amb a 1* ^a^	Amb a 4	Amb a 6			*Amb a 11*
*Ambrosia artemisiifolia*				Amb a 10										
Mugwort	7–9		Art v 4	Art v 5					Art v 6	*Art v 1* ^a^	Art v 3^a^			
*Artemisia vulgaris*														
Feverfew	7-9									Par h 1				
*Parthenium hysterophorus*														
Pellitory	all season		Par j 3	Par j 4							*Par j 1*			
*Parietaria judaica*											*Par j 2* ^a^			
English plantain	4–9											*Pla l 1* ^a^		
*Plantago lanceolata*														
Goosefoot	6–10		Che a 2	Che a 3								*Che a 1* ^b^		
*Chenopodium album*														
Russian thistle	7–9		Sal k 4									Sal k 5	*Sal k 1* ^*a*^	
*Salsola kali*														
Amaranth	7–9		Ama r 2											
*Amaranthus retroflexus*														
Annual mercury	5–10		*Mer a 1* ^b^											
*Mercurialis annua*														

Major allergens highlighted in italics

^a^Commercially available for single- and multiplex analysis

^b^Commercially available for multiplex analysis only

^c^Commercially available for singleplex only

^d^Allergen not officially acknowledged by the WHO/IUIS allergen nomenclature sub-committee

^e^Sequence homology to N-terminus of Ole e 9, listed as carbohydrate-binding molecule

## Methods for Molecule-Based Allergy Diagnosis

Apart from clinical history, skin prick tests (SPT) using allergen extracts represent one of the most common used in vivo diagnostic tools to confirm an immediate IgE-mediated allergic reaction. Since the 1990s, the performance of several purified recombinant pollen allergens was investigated in provocation tests and compared to natural allergens and extracts [13•]. Though refinement of diagnosis with purified molecules was proven effective, in vivo diagnosis using components is nowadays widely restricted to GMP-produced material. Alternatives such as the basophil activation test (BAT), an in vitro method to monitor upregulation of activation markers CD63 and CD203 upon allergen-triggered activation of primary basophils, or mediator release assays with passively sensitized basophils, which measure either histamine or β-hexosaminidase, may help to circumvent this problem. While these assays demonstrate functional allergen responses, they are rather demanding regarding the costs and experimental skills and thus are not used in daily practice. Measurement of specific IgE (sIgE) in serum is currently the most widely used in vitro method for allergy diagnosis. Besides allergen extracts provided for singleplex analyses (ImmunoCAP), a panel of purified natural and recombinant components is commercially available for routine diagnosis. In addition, allergen microarrays with more than 100 purified allergen molecules (ImmunoCAP ISAC) enable simultaneous IgE measurement using only minute amounts of blood. These multiplex assays offer an attractive alternative to refine allergy diagnosis, monitor disease progression as well as therapeutic outcomes [14]. All in vitro methods provide reliable information on IgE sensitization while correlation with clinically relevant symptoms remains challenging [15].

## Grass Pollen Allergens

Grasses are cultivated for food, animal fodder, surface vegetation, and meadows, but also represent one of the major causes of respiratory allergies worldwide [16–19]. The clinically most relevant source of pollen allergens is found within the Poaceae family. Among this, the Pooideae subfamily is prevalent in temperate climates and the most abundant allergenic species within the Pooideae are Timothy grass, Perennial ryegrass, Orchard grass, and Kentucky blue grass. The pollination season typically starts in spring and lasts until late summer, pollen peaks are usually found between June and July. In subtropical regions, members of the Chloridoideae and Panicoideae subfamilies are widely distributed. Here, the most important species triggering pollen allergy are Bermuda grass, Bahia grass, and Johnson grass [20]. Pollination of these grasses persists throughout the year peaking from early summer through autumn. Climatological changes due to global warming and human activities have additionally influenced pollen distribution as well as potency which further increases sensitization risks [21••].

### Timothy Grass

Timothy grass is widely distributed in the temperate climate and one of the best characterized allergenic grasses. The highest sensitization prevalence is registered in the European population, ranging from 18.5 to 28.5 % [6]. A prevalence of 36 % among Austrian pupils was recently found using allergen microarray analysis (Stemeseder et al., unpublished). The majority of commercially available components for grass pollen allergy originate from Timothy grass (Table 1). In this context, Phl p 1, a major allergen from Timothy grass with a sensitization prevalence of >90 % among grass pollen allergic patients, seems extremely important [22, 23]. Phl p 1-specific IgE is considered a marker for genuine sensitization and was recently also proposed as “initiator molecule” for Timothy grass pollen allergy [21••, 24••]. In the study by Hatzler et al., 75 % of grass pollen allergic patients started with mono-sensitization to Phl p 1, in some cases years before onset of clinical symptoms, which subsequently developed into poly-sensitization towards other grass pollen allergens [24••].

The second major allergen in this source is Phl p 5, triggering specific IgE in 65–90 % of grass pollen allergic patients. Together with Phl p 1, it is considered a marker molecule for grass pollen allergy, and in rare cases of absent Phl p 1-specific IgE, Phl p 5 is giving reliable results of genuine sensitization [21••, 22]. Phl p 5 is highly cross-reactive with other group 5 grass pollen allergens, which are restricted to grasses of the Pooideae subfamily [25•, 26]. A high sequence homology with Phl p 6 is observed, and significant levels of IgE cross-reactivity have been demonstrated [26]. Phl p 6 is frequently used together with Phl p 2 and Phl p 5 to confirm specific sensitization to Pooideae grasses [21••]. However, care should be taken with interpretation of Phl p 2 results since a homologous molecule termed Sor h 2 was recently identified in Johnson grass, a member of the Panicoideae subfamily [25•]. The tentatively termed Timothy grass pollen allergen Phl p 3 shows sequence identity with Phl p 2 and partially with the C-terminus of Phl p 1; however, while cross-reactivity between group 2 and 3 grass pollen allergens was demonstrated, relevant cross-reactivity to group 1 allergens is not evident [27].

Among grass pollen components, solely Phl p 4 is provided as natural protein harboring CCDs. Recombinant Phl p 4 demonstrates lower IgE reactivity, thus IgE-binding to commercially available natural Phl p 4 should be analyzed for CCDs reactivity [7, 28]. In Timothy grass, the panallergens Phl p 7 (polcalcin) and Phl p 12 (profilin) are present probably inducing IgE cross-reactivity with homologs from trees, weeds and foods [29]. In the absence of Phl p 1- and/or Phl p 5-specific IgE, sensitization to panallergens is not indicative of genuine grass pollen reactivity [21••]. Nevertheless, an association of the Phl p 12 sensitization and the development of oral allergy syndromes were demonstrated in Italian children [30]. Due to the low sequence similarity, IgE cross-reactivity of Phl p 11 with other Ole e 1-like family members from trees and weeds is absent or very limited [29] (Stemeseder et al., unpublished).

### Perennial Ryegrass, Orchard Grass, and Kentucky Blue Grass

Allergens from Perennial ryegrass, Orchard grass, and Kentucky blue grass have been identified and are listed in Table 1, but none of those is commercially available for diagnosis. Due to high sequence identity and extensive IgE cross-reactivity of allergens within the Pooideae subfamily, diagnosis to temperate grasses is usually performed with components from Timothy grass [21••, 25•, 26, 31].

### Bermuda Grass

Cyn d 1, the major allergen from Bermuda grass, is currently the only commercially available molecule of the subtropical grasses. It is considered as biomarker for genuine sensitization to the Chloridoideae subfamily [21••]. Cyn d 1 is recognized by 76–100 % of grass pollen allergic patients, which may also result in IgE cross-reactivity with Phl p 1 [31]. Therefore, it was suggested that the primary sensitization to Bermuda grass is indicated when specific IgE-levels to Cyn d 1 exceed IgE-binding to Phl p 1 and IgE to Phl p 5 is not detectable. Since Cyn d 1 is harboring CCDs, glycan-derived reactivity should be monitored during diagnosis [7].

### Bahia Grass and Johnson Grass

Currently, no allergen from the Panicoideae subfamily is commercially available for molecular diagnosis. In recent years, a growing number of allergens from these sources have been described. Timbrell et al. showed improved sensitivity and specificity when using nPas n 1 for molecular diagnosis of Bahia grass pollen allergic patients [32•]. The authors suggested to include the Pas n 1 for commercially available diagnosis of Bahia grass allergy as a sub-group of patients showed species-specific IgE and T cell reactivity [33, 34]. As mentioned above, Sor h 2 was recently identified in Johnson grass which refutes the assumption that group 2 allergens are absent in subtropical grasses [21••, 25•, 35].

## Tree Pollen Allergens

In general, trees are woody perennial plants with an elongated self-supporting stem or trunk and supporting branches that form a more or less defined crown. To distinguish trees from shrubs, a certain stem height or diameter is sometimes used as decision criteria. Trees do not comprise a single taxonomic group and include various species that separately evolved stems and branches [36]. Most of the clinically relevant pollen allergens are produced by wind-pollinated trees belonging to only four different orders, which show an almost worldwide distribution. For details on tree pollen allergens, review Asam et al. [37••].

### Birch and Related Fagales Species

Classified within the order of Fagales, birch and the related tree species alder, hornbeam, hop-hornbeam, hazelnut, beech, chestnut, and oak constitute the main cause of early seasonal rhinitis in the temperate climate zone of the Northern Hemisphere [8]. Worldwide, more than 100 million patients suffer from birch pollen allergies and in Europe clinically relevant sensitization to birch affects around 19.6 % of the allergic population. Clinically relevant sensitization rates to hazel and alder pollen were reported at 17.1 and 16.2 %, respectively [38••, 39]. The major allergens from Fagales trees are classified as members of the pathogenesis-related-10 (PR-10) proteins, and Bet v 1 from birch pollen is generally acknowledged as main sensitizer and marker allergen of this family (Table 2). Among birch pollen allergic individuals, sensitization rates to Bet v 1 of up to 90 % or more have been reported [40, 41]. However, inhibition experiments revealed that besides birch, several other Fagales species might have the potential to sensitize susceptible individuals [9]. This seems especially important in areas where birch trees are virtually absent, thus exposure to other allergenic Fagales pollen will eventually lead to sensitization and the development of Fagales pollen allergy. Currently, Bet v 1 is the only group 1 Fagales allergen offered for singleplex, while Aln g 1 and Cor a 1 are additionally available for multiplex analysis. Since oak populations are very wide-spread not only in Europe but also in vast parts of North America and Asia and do not necessarily coexist with birch or hazel, Que a 1 should be considered for diagnosis especially in those areas. More than 70 % of birch pollen allergic patients develop adverse symptoms to food such as fruits, nuts, or vegetables [42]. This clinical condition often referred to as pollen-food syndrome always involves a pre-sensitization to Fagales pollen allergens [43]. Therefore, diagnosis of Bet v 1-related food allergies is directly linked to a correct diagnosis of the underlying pollen allergy. Moreover, several minor allergens, among them the panallergens profilin and polcalcin, have been identified in many allergenic Fagales species, evoking sensitization rates of 44.6 and 9.4 %, respectively [44].

### Plane Tree

Plane trees belong to the order of Proteales and are native to the Northern Hemisphere. They preferentially grow in temperate regions from Asia to Europe and North America. Due to their resistance towards pollution, they are widely used as ornamental trees in urban areas and at roadsides [45, 46]. With 3.3 % in the allergic population, clinically relevant sensitization throughout Europe is rather low but can reach almost 12 % in the UK [38••]. More than 80 % of plane-sensitized patients react to the major allergens Pla a 1 (plant invertase inhibitor) and Pla a 2 (polygalacturonase) [45, 47, 48]. The minor allergen Pla a 3 is a member of the nsLTP family and shows high cross-reactivity with Pru p 3, the major allergen from peach [49].

### Olive, Ash, Lilac, and Privet

The major allergy-eliciting species within the order of Lamiales are olive, ash, lilac, and privet. Olive trees are preferentially cultivated in the Mediterranean area where up to 70 % of patients with respiratory symptoms are sensitized to olive pollen [8]. With a sensitization prevalence of 80 % among olive pollen allergic patients, Ole e 1 represents the major allergen and thus is the eponym of the Ole e 1-like protein family with homologs in tree, grass, and weed pollen. While cross-reactivity to homologues in other Oleaceae trees is very high, cross-reactivity with grass (Phl p 11) and weed (e.g., Pla l 1) Ole e 1-like allergens is limited [37••] (Stemeseder et al., unpublished). Besides Ole e 1, 10 other olive pollen allergens, including the panallergens Ole e 2 (profilin) and Ole e 3 (polcalcin), have been described and details can be reviewed in Villalba et al. [50•]. Depending on geographic location and thus on the exposure level of the population, the allergens Ole e 6, 7, 10, and 11 may eventually become major allergens of olive pollen [50•, 51].

In central Europe, ash pollen may cause sensitization rates from 4 to 18 % [37••]. The major allergen of European ash, Fra e 1, has been classified as member of the Ole e 1-like family and shows high sequence similarity to other Ole e 1-like proteins from Lamiales [52]. A recent study from Imhof et al. confirmed the high cross-reactivity between Ole e 1 and Fra e 1 and suggested using Ole e 1 as diagnostic maker of ash pollen allergy [53]. Moreover, a β-1,3-glucanase from ash termed Fra e 9 was recently identified, which can account for a sensitization prevalence up to 60 % in distinct areas of France [54]. Common privet as well as lilac are often used for ornamental purposes and frequently found in Europe, Asia, and North America. Both express highly cross-reactive major allergens belonging to the Ole e 1-like protein family (Lig v 1 and Syr v 1, respectively); sensitization rates are however generally low [55].

### Allergenic Cypress Species, Japanese Cedar, and Juniper Trees

The order of Pinales comprises allergenic cypress, cedar as well as juniper species. In contrast to the previously described allergenic trees, Pinales are gymnosperms widely distributed throughout the Northern Hemisphere. Mediterranean cypress and its American relative, Arizona cypress, which is primarily native to the south-west of North America, are closely related to evergreen trees which coexist in the Mediterranean area (www.eol.org). Among the allergic individuals in Europe, 2.6 % show a clinically relevant sensitization to cypress, which is generally low, however, may reach levels of up to 42.7 % as reported for allergic patients in Italy [56]. The major allergens of both species, Cup s 1 and Cup a 1, belong to the pectate lyase family and share 95.1 % sequence identity. Moreover, clinically relevant, highly cross-reactive, allergenic pectate lyases have been identified as major allergens in Mountain cedar (Jun a 1) endemic in the USA, as well as in Japanese cypress (Cho o 1) and Japanese cedar (Cry j 1). The latter two species form dominant populations on the Japanese island [37••]. Sensitization rates evoked by Cha o 1 or the more potent Cry j 1 affect up to 40 % of the population in certain areas of Japan [57, 58], whereas sensitization to Jun a 1 represents a serious health risk in Texas and Mexico [59]. Moreover, the polygalacturonases Jun a 2, Cry j 2 and Cha o 2, have been identified as major allergens in these Cupressaceae species being responsible for sensitization rates of up to 80 % [37••, 60, 61]. Nevertheless, allergenic polygalacturonases did not get much attention so far but should eventually be considered as candidates for allergy diagnosis.

## Weeds Pollen Allergens

The term “weed” does not refer to any specific botanical family but rather describes plants outside the order of trees or grasses. They are used as culinary herbs, medicinal plants, and are frequently ecologically adaptive segetal plants [62]. Climatic changes are generally impacting the flora, which might be particularly advantageous for weeds as they are able to dominate groundcover, adapt to various environments, or reside in ecologic niches. Weed pollen allergic patients are frequently poly-sensitized to diverse plant sources, thus molecule-based approaches are especially valuable for precise diagnosis. A comprehensive overview on production and botanical classification of weed pollen allergens is provided in Gadermaier et al. and Villalba et al. [63, 64•].

### Ragweed

Short ragweed is native to Northern and Central America, where it is one of the major elicitors of type I pollen allergy. Sensitization prevalence can be up to 15.3 % in the general population and the weed demonstrates high cross-reactivity with other *Ambrosia* spp. [65–67]. Since its introduction as ballast grain, ragweed also became a relevant allergen source in parts of Europe, Asia, and Australia [5, 68]. Global warming already substantially prolonged the ragweed pollen season and further spreading of the weed to the North is predicted by in silico models [69, 70].

The pectate lyase Amb a 1 is highly abundant in ragweed pollen and represents the dominant allergen. Based on a sensitization prevalence of >95 %, the natural allergen is currently considered the marker allergen for genuine ragweed sensitization [63, 71]. In contrast to lacking cross-reactivity to homologs from the Cupressaceae family, antibody cross-reactivity with Art v 6 from mugwort pollen is observed. However, inhibition assays and T cell studies primarily point at a stronger allergenic potential and sensitizing role of Amb a 1 [72, 73]. In a recent study, primary sensitizing capacity of Art v 6 was also demonstrated and the authors concluded that true co-sensitization of ragweed and mugwort is generally rare [10].

Recently, a cysteine protease with structural homology to group I allergens from house dust mites was identified in ragweed and designated Amb a 11 [74••]. It represents also a major allergen since 66 % of patients reacted to the molecule, which was previously “hidden” in the Amb a 1 fraction. Though the majority of allergenic reactivity is clearly attributed to Amb a 1 and isolated Amb a 11 reactivity seems rare, it could be relevant for increasing the diagnostic panel [74••]. The minor ragweed allergen Amb a 4 consists of a defensin-like domain fused with a proline-rich region with homology and IgE cross-reactivity to Art v 1 from mugwort pollen [75]. Ragweed allergens belonging to the profilin, polcalcin, and nsLTP families (Table 2) are accounting for moderate to low IgE reactivity [63]. Recent transcriptome and immunoproteome data suggest 68 and 41 % IgE reactivity for ragweed polygalacturonase and enolase, respectively [71], which together with Amb a 3 might be worth investigating at the molecule level.

### Mugwort

Common mugwort is endemic in the Northern Hemisphere and Australia and represents the best studied plant within the genus. Among pollinosis patients, sensitization to mugwort pollen ranges between 10 and 14 % in the European and Asian population [63, 76]. Habitat and pollination of mugwort are vastly overlapping with ragweed and thus confounding allergy anamnesis. Art v 1, consisting of a defensin-like domain fused with a proline-rich region, represents the major allergen with a sensitization prevalence of 70–95 % [77, 78]. The marker allergen demonstrates partial IgE cross-reactivity with Amb a 4 from ragweed and Par h 1 from feverfew pollen. Primary sensitization seems to be predominately evolving by the major mugwort allergen (Pablos et al., unpublished data). In addition, Art v 3 is of particular interest in regions where nsLTP-related food and pollen allergies are observed [79]. While Art v 3 might pave the way to food-related symptoms in peach allergic patients [80], recent data also suggest that it is able to *bona fide* elicit respiratory symptoms in patients [81, 82•]. In mugwort sensitized patients, Art v 6 in hand with Art v 1 is considered highly indicative for primary mugwort sensitization [10].

### Pellitory

Pollen of pellitory is responsible for allergic reactions predominately in Mediterranean regions with sensitization frequencies reaching 60–90 % [63]. Due to climatic changes, flowering periods throughout the year were registered [83]. The main allergen components are Par j 1 and Par j 2, both belonging to the nsLTP family showing a sensitization frequency of up to 95 % [63, 84]. They are considered marker allergens for genuine pellitory allergy, since they do not cross-react with homologs from other pollen and food [85]. Par j 2 showed high sensitivity and specificity and is currently used for routine molecule-based diagnosis of pellitory [86].

### English Plantain

*Plantago* spp. are worldwide abundant weeds showing recurrent flowering seasons overlapping with grasses. Studies demonstrated a high clinical relevance in parts of southern and central Europe [87•, 88]. The major allergen Pla l 1 belongs to the Ole e 1-like family and is recognized by >90 % of patients [63, 87•]. Since IgE cross-reactivity with other family members is limited, it represents a highly specific marker for molecule-based diagnosis [62] (Stemeseder et al., unpublished).

### Russian Thistle and Goosefoot

These weeds are considered invasive species found in arid regions of the Northern Hemisphere and Australia [62, 64•]. Due to their previous use in greening programs, they are highly abundant in Middle Eastern countries where they can represent major sensitizers for rhinitis and asthma [64•, 89, 90]. Amaranthaceae pollen also gained allergological relevance in areas of Spain, and they are expected to play an increasingly important role [91, 92]. The pectin methylesterase Sal k 1 accounts for the majority of IgE reactivity to Russian thistle and was shown to be a marker for genuine sensitization as it allows discrimination from chenopod sensitization [93, 94]. Natural glycosylated Sal k 1 is currently available for molecule-based diagnosis; however, the use of a recombinant molecule might be superior as CCDs can be avoided [95]. Sal k 5, a member of the Ole e 1-like protein family showed 30 % sensitization prevalence and considerable high IgE cross-reactivity with Che a 1 from chenopod [96]. Notably, this IgE reactivity seems specific for Amaranthaceae and not related to Ole e 1 from olive pollen [96].

Che a 1 belongs to the Ole e 1-like family and presents a sensitization frequency of >70 % among chenopod allergic patients [89, 97]. High IgE reactivity to chenopod profilin and polcalcin is noted, and diagnostic specificity was increased by using a cocktail of three purified proteins [89]. However, the high IgE cross-reactivity of the panallergens rather suggests that Che a 1 should be primarily considered for diagnosis although discrimination from Russian thistle sensitization seems challenging [64•, 98].

### Annual Mercury

Annual mercury was reported to be an important source of pollinosis in distinct regions of Mediterranean countries [99]. For diagnostic purpose, the profilin Mer a 1 is currently available in multiplex assays; however, due to high IgE cross-reactivity with other pollen profilins, interpretation of these results might be limited.

## Pollen Panallergens

The term “panallergen” refers to the ubiquitous distribution of these allergens in diverse sources and profilins and polcalcins are prototypic examples [100]. The clinical relevance is considered limited, but the broad IgE reactivity highly influences extract-based diagnosis [92, 101••]. A panel of profilins and polcalcins, considered representatives of the entire panallergen group, is available for molecule-based diagnosis allowing discrimination between genuine and panallergen sensitization (Tables 1 and 2). For in vivo diagnosis, natural panallergens from palm tree were previously used [102]. In contrast, members of the pectate lyase, nsLTP, and Ole e 1-like families demonstrate heterogeneous reactivity pattern. IgE cross-reactivity is mostly confined to closely related molecules with high sequence identity and might vary in different populations (Fig. 1).

## Conclusions

Diagnosis of pollen allergies mainly relies on careful anamnesis, which includes narrowing down the sensitizing plant by the time clinical symptoms are observed. However, flowering periods are frequently overlapping and can vary in distinct geographic regions while climatic changes are expected to further aggravate the problem [1, 69, 70]. Pollen allergic patients are typically reacting to more than one source, thus specific diagnosis is applied either using allergen extracts or purified components. Based on molecule-based approaches, more specific results are obtained and clinically irrelevant sensitizations due to, e.g., panallergens or other IgE cross-reactive compounds can be circumvented [11]. The currently available panel of allergen components is covering most of the common allergen sources; however, in comparison to extract-based products, there is a substantial gap in quantity. Allergy research will certainly further increase the panel of relevant allergens of known sources as well as novel allergens from plants that were less explored for diagnosis [37••]. Molecule-based diagnosis can improve sensitivity, specificity, predict severity of reactions, and identify the genuine sensitizing source [103]. This refined diagnosis was recently shown to change a large proportion of allergen immunotherapy prescription as opposed to relying on anamnesis and SPT alone [101••]. In future, allergy diagnosis using reliable biomarkers might be crucial when considering allergen immunotherapy in the context of precision medicine [104].
